# Data on physical and electrical properties of (ZrO_2_)_1-x_(Sc_2_O_3_)_x_(CeO_2_)_y_ and (ZrO_2_)_1-x-y-z_(Sc_2_O_3_)_x_(CeO_2_)_y_(Y_2_O_3_)_z_ solid solution crystals

**DOI:** 10.1016/j.dib.2019.104061

**Published:** 2019-05-25

**Authors:** M.A. Borik, A.S. Chislov, A.V. Kulebyakin, I.E. Kuritsyna, V.A. Kolotygin, E.E. Lomonova, F.O. Milovich, V.A. Myzina, N.Yu. Tabachkova

**Affiliations:** a Prokhorov General Physics Institute, Russian Academy of Sciences, Vavilov Str. 38, 119991 Moscow, Russia; b National University of Science and Technology (MISIS), Leninskiy prospekt 4, 119049 Moscow, Russia; c Institute of Solid State Physics, Russian Academy of Sciences, Academician Osip'yan Str. 2, 142432 Chernogolovka, Moscow District, Russia

**Keywords:** Single crystals, Solid oxide fuel cell, Solid solutions, Ionic conducting materials, ZrO_2_–Sc_2_O_3_-CeO_2_

## Abstract

The data presented in this article are related to the research article entitled “Phase stability and transport characteristics of (ZrO_2_)_1-x_(Sc_2_O_3_)_x_(СeO_2_)_y_ and (ZrO_2_)_1-x-y-z_(Sc_2_O_3_)_x_(СeO_2_)_y_(Y_2_O_3_)_z_ solid solution crystals” https://www.sciencedirect.com/science/article/pii/S2352340917302329 [1]. It contains data on densities and microhardness of the as-grown crystals. The data on the specific conductivity of the as-grown and annealing at 1000 °С for 400 h ScCeSZ and ScCeYSZ crystals in the temperature range 623–1173 K is also included in this article. The article describes also the growth of the (ZrO_2_)_1-x_(Sc_2_O_3_)_x_(СeO_2_)_y_ and (ZrO_2_)_1-x-y-z_(Sc_2_O_3_)_x_(СeO_2_)_y_(Y_2_O_3_)_z_ solid solution crystals using directional melt crystallization in a cold crucible.

**Table:** Specifications Table

Subject area	*Materials Science*
More specific subject area	*Solid state electrolyte*
Type of data	*Table, graph*
How data was acquired	*Hydrostatic weighing - Sartorius hydrostatic balance (Switzerland)* *The microhardness - DM 8 В AUTO microhardness tester (Affri, Italy) with a 50 g load.* *The impedance spectroscopy - Solartron SI 1260 frequency analyzer (Solartron Analytical, United Kingdom)*
Data format	*Raw, filtered and analyzed.*
Experimental factors	*The crystals were annealed in a Supertherm HT04/16 high-temperature resistance furnace in air at 1000 °C for 400 h.*
Experimental features	*All crystals were grown by directional melt crystallization in a cold crucible*[2]
Data source location	*Moscow, Russia*
Data accessibility	*Data are available with this paper*
Related research article	*D.A. Agarkov, M.A. Borik, V.T. Bublik, A.S. Chislov, A.V. Kulebyakin, I. E. Kuritsyna, V.A. Kolotygin, E.E. Lomonova, F.O. Milovich, V.A. Myzina, V.V. Osiko, N.Yu. Tabachkova. Phase stability and transport characteristics of (ZrO*_*2*_*)*_*1-x*_*(Sc*_*2*_*O*_*3*_*)*_*x*_*(СeO*_*2*_*)*_*y*_*and (ZrO*_*2*_*)*_*1-x-y-z*_*(Sc*_*2*_*O*_*3*_*)*_*x*_*(СeO*_*2*_*)*_*y*_*(Y*_*2*_*O*_*3*_*)*_*z*_*solid solution crystals. J. Alloy. Compd. 791 (2019) 445–451.*[1]

**Table:** 

**Value of the data** • The data on oxygen/ionic conductivity of the (ZrO_2_)_1-x_(Sc_2_O_3_)_x_(СeO_2_)_y_ and (ZrO_2_)_1-x-y-z_(Sc_2_O_3_)_x_(СeO_2_)_y_(Y_2_O_3_)_z_ solid solution crystals is very useful for development solid state electrolytes for SOFCs. • The data on high-temperature degradation of conductivity to get more depth information about ionic conduction mechanism in solid state electrolytes. • The present data could be helpful for researchers involved in the crystal growth of the high temperature materials.

## 1 Data

**Fig. 1:**
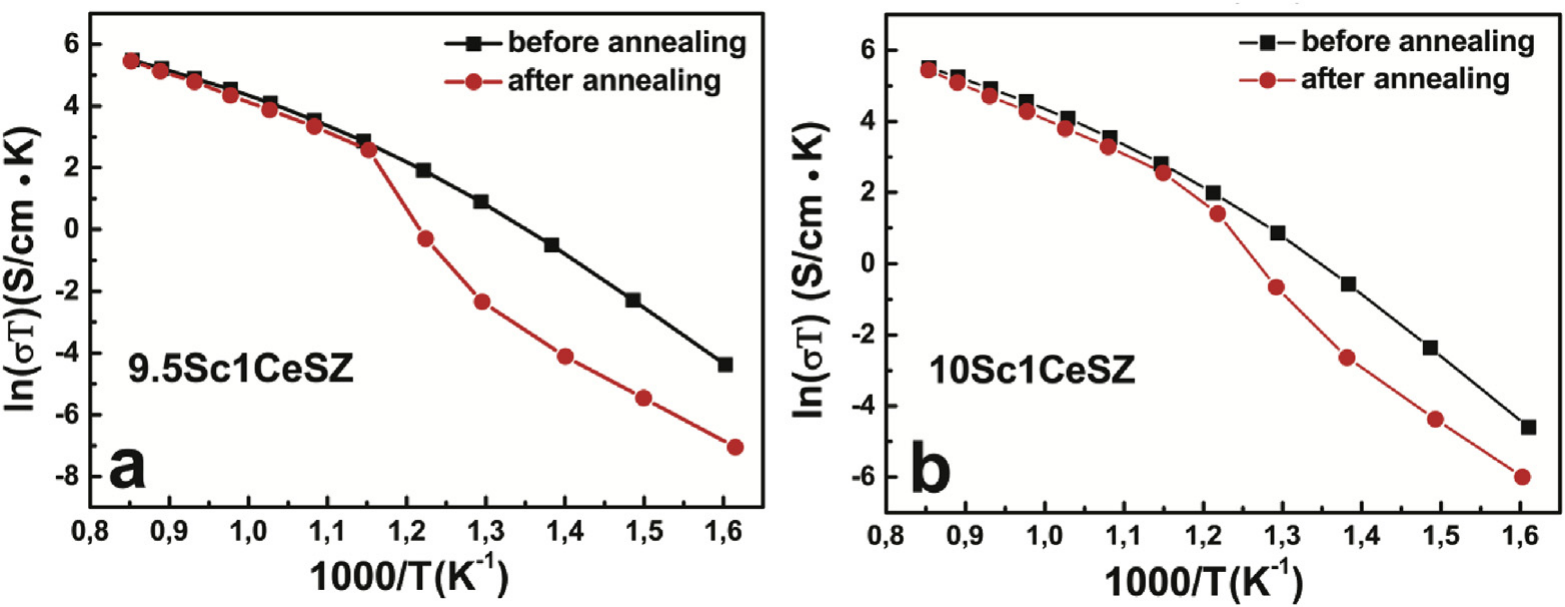
Arrhenius plot of specific bulk conductivity of as-grown and as-annealed crystals ScCeSZ.

**Fig. 2:**
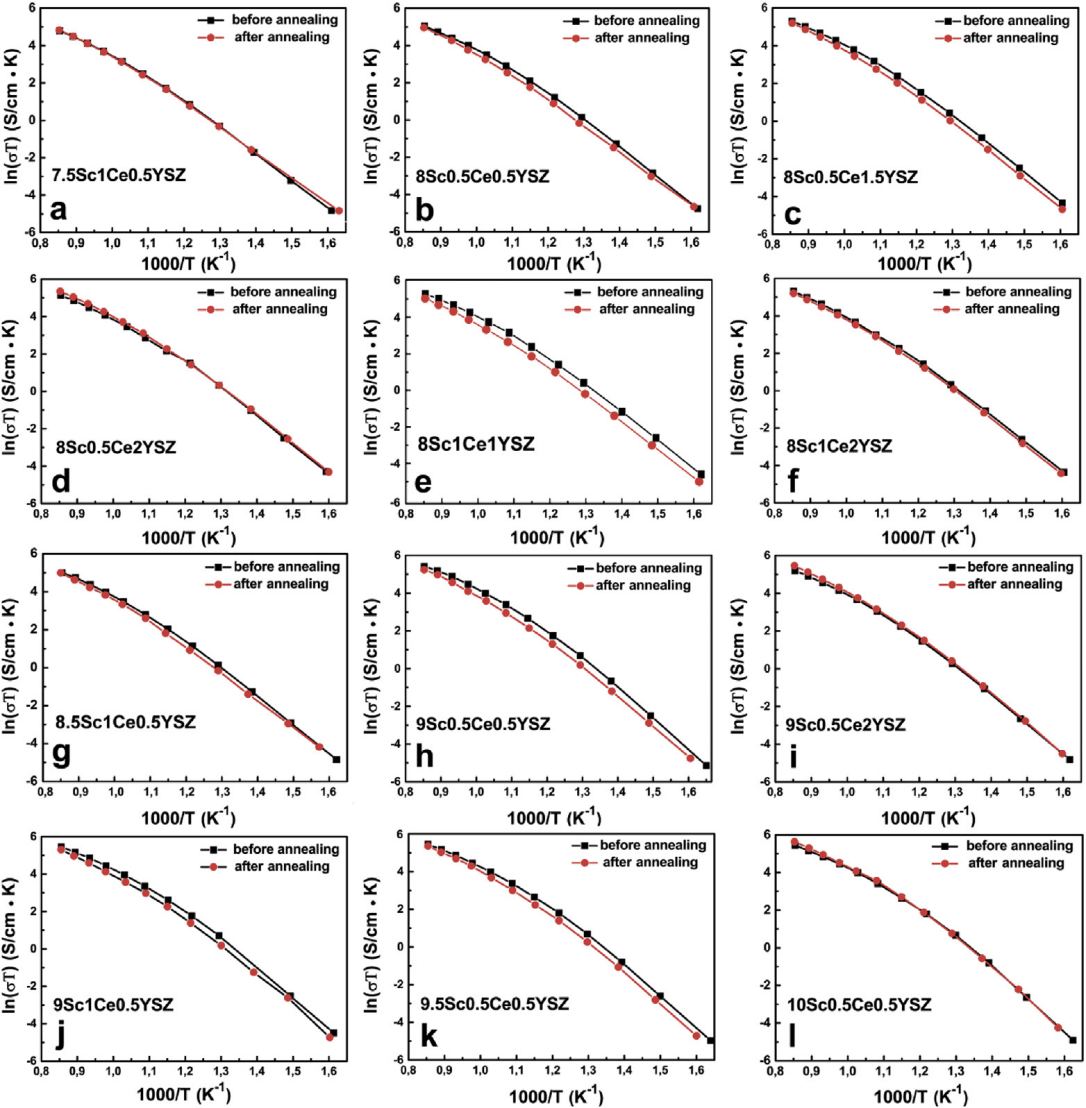
Arrhenius plot of specific bulk conductivity of as-grown and as-annealed ScCeYSZ crystals.

This dataset contains information about density, microhardness and specific conductivity of the scandia- ceria- and yttria-stabilized zirconia. Table 1 shows the chemical composition, brief notations, densities and microhardness of the as-grown crystals used in the further analysis. Table 2 shows the specific conductivity of the as-grown and annealing at 1000 °С for 400 h ScCeSZ and ScCeYSZ crystals in the temperature range 973–1173 K. Arrhenius plot of specific bulk conductivity of as-grown and as-annealed crystals ScCeSZ is shown in Fig. 1. The same plot for as-grown and as-annealed crystals ScCeYSZ is shown in Fig. 2.

**Table 1:** Chemical composition, brief notations, densities and microhardness of the as-grown crystals. Part of the data is already published in Ref. [1].

Chemical composition	Notations	Density, g/cm^3^	Microhardness, Hv, kg/mm^2^
ScCeSZ			
(ZrO_2_)_0.90_(Sc_2_O_3_)_0.085_(CeO_2_)_0.015_	8.5Sc1.5CeSZ	5.787 ± 0.001	1560 ± 50
(ZrO_2_)_0.90_(Sc_2_O_3_)_0.09_(CeO_2_)_0.01_	9Sc1CeSZ	5.791 ± 0.004	1680 ± 20
(ZrO_2_)_0.90_(Sc_2_O_3_)_0.095_(CeO_2_)_0.005_	9.5Sc0.5CeSZ	5.778 ± 0.004	1585 ± 50
(ZrO_2_)_0.89_(Sc_2_O_3_)_0.10_(CeO_2_)_0.01_	10Sc1CeSZ	5.757 ± 0.004	1720 ± 20
(ZrO_2_)_0.895_(Sc_2_O_3_)_0.095_(CeO_2_)_0.01_	9.5Sc1CeSZ	5.757 ± 0.004	1690 ± 60
ScCeYSZ			
(ZrO_2_)_0.91_(Sc_2_O_3_)_0.075_(CeO_2_)_0.01_(Y_2_O_3_)_0.005_	7.5Sc1Ce0.5YSZ	5.835 ± 0.003	1679 ± 47
(ZrO_2_)_0.91_(Sc_2_O_3_)_0.08_(CeO_2_)_0.005_(Y_2_O_3_)_0.005_	8Sc0.5Ce0.5YSZ	5.829 ± 0.001	1760 ± 32
(ZrO_2_)_0.90_(Sc_2_O_3_)_0.08_(CeO_2_)_0.01_(Y_2_O_3_)_0.01_	8Sc1Ce1YSZ	5.812 ± 0.001	1610 ± 40
(ZrO_2_)_0.90_(Sc_2_O_3_)_0.08_(CeO_2_)_0.005_(Y_2_O_3_)_0.015_	8Sc0.5Ce1.5YSZ	5.841 ± 0.004	1570 ± 40
(ZrO_2_)_0.90_(Sc_2_O_3_)_0.85_(CeO_2_)_0.01_(Y_2_O_3_)_0.005_	8.5Sc1Ce0.5YSZ	5.801 ± 0.003	1575 ± 30
(ZrO_2_)_0.90_(Sc_2_O_3_)_0.09_(CeO_2_)_0.005_(Y_2_O_3_)_0.005_	9Sc0.5Ce0.5YSZ	5.785 ± 0.002	1560 ± 80
(ZrO_2_)_0.895_(Sc_2_O_3_)_0.095_(CeO_2_)_0.005_(Y_2_O_3_)_0.005_	9.5Sc0.5Ce0.5YSZ	5.767 ± 0.001	1640 ± 50
(ZrO_2_)_0.885_(Sc_2_O_3_)_0.009_(CeO_2_)_0.005_(Y_2_O_3_)_0.02_	9Sc0,5Ce2YSZ	5.755 ± 0.003	1640 ± 50
(ZrO_2_)_0.895_(Sc_2_O_3_)_0.08_(CeO_2_)_0.005_(Y_2_O_3_)_0.02_	8Sc0.5Ce2YSZ	5.829 ± 0.001	1630 ± 20
(ZrO_2_)_0.895_(Sc_2_O_3_)_0.9_(CeO_2_)_0.01_(Y_2_O_3_)_0.005_	9Sc1Ce0.5YSZ	5.782 ± 0.002	1580 ± 50
(ZrO_2_)_0,89_ (Sc_2_O_3_)_0.08_(CeO_2_)_0.01_(Y_2_O_3_)_0.02_	8Sc1Ce2YSZ	5.831 ± 0.001	1550 ± 40
(ZrO_2_)_0.89_(Sc_2_O_3_)_0.1_(CeO_2_)_0.005_(Y_2_O_3_)_0.005_	10Sc0.5Ce0.5YSZ	5.755 ± 0.001	1830 ± 40

**Table 2:** The specific conductivity of the as-grown and annealing ScCeSZ and ScCeYSZ crystals in the temperature range 973–1173 K.

Sample	Conductivity, S/cm (as grown)				Conductivity, S/cm (annealing 1000 °С/400 h)			
	973К	1073 К	1123 К	1173 К	973К	1073 К	1123 К	1173 К
8.5Sc1.5CeSZ(10)	0.056	0.119	0.157	0.197	0.039	0.087	0.121	0.161
9Sc1CeSZ(10)	0.054	0.113	0.143	0.164	0.038	0.086	0.120	0.158
9.5Sc0.5CeSZ(10)	0.064	0.134	0.175	0.216	0.047	0.107	0.149	0.195
9.5Sc1CeSZ(10.5)	0.062	0.124	0.164	0.205	0.049	0.112	0.149	0.199
10Sc1CeSZ(11)	0.062	0.128	0.170	0.212	0.045	0.103	0.144	0.197
7.5Sc1Ce0.5YSZ	0.024	0.056	0.080	0.101	0.023	0.056	0.081	0.106
8Sc0.5Ce0.5YSZ(9)	0.034	0.076	0.102	0.133	0.026	0.066	0.091	0.123
8Sc0.5Ce1.5YSZ(10)	0.045	0.100	0.133	0.170	0.033	0.081	0.116	0.158
8Sc1Ce1YSZ(10)	0.045	0.095	0.136	0.171	0.027	0.070	0.097	0.132
8.5Sc1Ce0.5YSZ	0..033	0.075	0.103	0.129	0.033	0.074	0.102	0.125
9Sc0.5Ce0.5YSZ(10)	0.055	0.121	0.159	0.193	0.037	0.090	0.128	0.161
8Sc0.5Ce2YSZ(10.5)	0.036	0.083	0.112	0.144	0.041	0.098	0.135	0.176
9Sc1Ce0.5YSZ(10.5)	0.055	0.120	0.158	0.204	0.038	0.093	0.127	0.176
9.5Sc0.5Ce0.5YSZ(10.5)	0.055	0.120	0.159	0.200	0.042	0.101	0.136	0.180
8Sc1Ce2YSZ(11)	0.039	0.095	0.130	0.170	0.034	0.081	0.115	0.152
10Sc0.5Ce0.5YSZ(11)	0.056	0.119	0.157	0.199	0.058	0131	0.182	0.239
9Sc0.5Ce2YSZ(11.5)	0.040	0.089	0.121	0.154	0.045	0.106	0.150	0.203

## 2 Experimental design, materials, and methods

All of the samples having nominal composition (ZrO_2_)_1-x_(Sc_2_O_3_)_x_(СeO_2_)_y_ (x = 0.085–0.10; y = 0.005–0.015) and (ZrO_2_)_1-x-y-z_(Sc_2_O_3_)_x_(СeO_2_)_y_(Y_2_O_3_)_z_ (x = 0.07–0.10; y = 0.005–0.010; z = 0.005–0.020) were prepared by directional melt crystallization in a cold crucible.

ZrO_2_, Sc_2_O_3_, СeO_2_, and Y_2_O_3_ powders of not less than 99.99 % purity grade were the initial materials. The crystallization of the melt was carried out in a water-cooled crucible 130 mm in diameter. The RF generator (frequency 5.28 MHz, maximum output power 60 kW) was used as a power source. The charge weight was 5 kg. The directional crystallization of the melt was achieved by moving the crucible with the melt downward relative to the induction coil at a 10 mm/h rate. The weight of the ingots was 3.5–4.0 kg. After the installation was shut down the ingot cooled down spontaneously. The cooling of the ingots was monitored by measuring the temperature on the surface of the upper heat screen with a Gulton 900–1999 radiation pyrometer (above 1000 °C) and a Pt/Pt-Rh thermocouple (1000 °C down to 500 °C). The average ingot cooling rate from the melt temperature to 1000 °C was 150–200 K/min and then down to 500 °C with 30 K/min. The process yielded ingots consisting of column crystals that could be mechanically separated into individual crystals. Typical dimensions of the crystals were 8–15 mm in cross-section and 30–40 mm in length.

The as grown crystals were then annealed in a Supertherm HT04/16 high-temperature resistance furnace in air at 1000 °C for 400 h.

The conductivity of the zirconia base crystals was measured in the 400–900 °C range using a Solartron SI 1260 frequency analyzer in the 1 Hz–5 MHz range. The resistivity was measured in a measurement cell using the four-probe method in a Nabertherm high temperature furnace (Nabertherm GmbH. Germany). The measurements were carried out on crystal plates size of 7 × 7 mm^2^ and thickness of 0.5 mm with symmetrically connected Pt electrodes. Platinum electrodes were annealed in air at the temperature 950 °C for 1 h. The ac amplitude applied to the sample was 24 mV. The impedance frequency spectrum was analyzed in detail using the ZView (ver.2.8) (Scribner Associates Inc., USA) software. The resistivity of the crystals was calculated based on the resultant impedance spectra and then the specific conductivities of the crystals were calculated taking into account the specimen dimensions.
