# Supplementary material for: Data on physical and electrical properties of (ZrO2)1-x(Sc2O3)x(CeO2)y and (ZrO2)1-x-y-z(Sc2O3)x(CeO2)y(Y2O3)z solid solution crystals
Source: Data Brief. 2019 May 25;25:104061. doi: 10.1016/j.dib.2019.104061 (PMC6562190; doi:10.1016/j.dib.2019.104061)
Supplement: Multimedia component 1 [file mmc1.docx]

Dear Editor!

We are hereby submitting a manuscript entitled “***Data on physical and electrical properties of (ZrO_2_)_1-x_(Sc_2_O_3_)_x_(СeO_2_)_y_ and (ZrO_2_)_1-x-y-z_(Sc_2_O_3_)_x_(СeO_2_)_y_(Y_2_O_3_)_z_ solid solution crystals***” by M.A. Borik, A.S. Chislov, A.V. Kulebyakin, I. E. Kuritsyna, V.A. Kolotygin, E.E. Lomonova, F.O. Milovich, V.A. Myzina,, N.Yu. Tabachkova for your consideration of its publication in Data in Brief.

The work described in the paper has not been published previously and it is not under consideration for publication elsewhere. Its publication is approved by all authors and tacitly or explicitly by the responsible authorities where the work has been carried out. If the paper is accepted for publication, it will not be published elsewhere in the same form, in English or in any other language, without the written consent of the Publisher.

Thank you for your kind consideration.

Best regards!
